# Perfusion Deficits and Functional Connectivity Alterations in Memory-Related Regions of Patients with Post-Traumatic Stress Disorder

**DOI:** 10.1371/journal.pone.0156016

**Published:** 2016-05-23

**Authors:** Yang Liu, Baojuan Li, Na Feng, Huangsheng Pu, Xi Zhang, Hongbing Lu, Hong Yin

**Affiliations:** 1 School of Biomedical Engineering, Fourth Military Medical University, Xi’an, Shaanxi, China; 2 Department of Physiology, Fourth Military Medical University, Xi’an, Shaanxi, China; 3 Department of Radiology, Xijing Hospital, Xi’an, Shaanxi, China; Central Institute of Mental Health, GERMANY

## Abstract

To explore the potential alterations in cerebral blood flow (CBF) and functional connectivity of recent onset post-traumatic stress disorder (PTSD) induced by a single prolonged trauma exposure, we recruited 20 survivors experiencing the same coal mining flood disaster as the PTSD (n = 10) and non-PTSD (n = 10) group, respectively. The pulsed arterial spin labeling (ASL) images were acquired with a 3.0T MRI scanner and the partial volume (PV) effect in the images was corrected for better CBF estimation. Alterations in CBF were analyzed using both uncorrected and PV-corrected CBF maps. By using altered CBF regions as regions-of-interest, seed-based functional connectivity analysis was then performed. While only one CBF deficit in right corpus callosum of PTSD patients was detected using uncorrected CBF, three more regions (bilateral frontal lobes and right superior frontal gyrus) were identified using PV-corrected CBF. Furthermore, the regional CBF of right superior frontal gyrus exhibited significantly negative correlation with the symptom severity (*r* = −0.759, *p* = 0.018). The resting-state functional connectivity analysis revealed increased connectivity between left frontal lobe and right parietal lobe. The results indicated the symptom-specific perfusion deficits and an aberrant connectivity in memory-related regions of PTSD patients when using PV-corrected ASL data. It also suggested that PV-corrected CBF exhibits more subtle changes that may be beneficial to perfusion and connectivity analysis.

## Introduction

Post-traumatic stress disorder (PTSD) is an anxiety disorder that develops after exposure to a terrifying event or ordeal in which grave physical harm occurred or was threatened [1]. Neuroimaging studies have identified a number of structural and functional alterations associated with PTSD. In these studies, an atrophy of the hippocampus [2, 3], as well as volumetric changes of the amygdala [4] and anterior cingulate gyrus [4] have been consistently reported. Meanwhile, functional neuroimaging studies have identified altered activations in the cingulate cortex [5], medial prefrontal cortex [6], and amygdala [6]. However, the majority of PTSD studies focused on subjects who experienced repeated and short-duration traumas, such as combat or abuse-related experience [1]. Few studies have investigated the effect of recent onset PTSD induced by a single prolonged trauma exposure. In previous studies, a series of volumetric and cortical thickness alterations were identified for this kind of PTSD [1, 3], without exploring possible alterations on perfusion and functional connectivity of the same subjects.

Currently, most perfusion studies on PTSD have been performed using positron emission tomography (PET) or single-photon emission computed tomography (SPECT) scans [7, 8]. Only two of them were based on magnetic resonance imaging (MRI), one using dynamic susceptibility contrast (DSC) perfusion to find relative cerebral blood flow (CBF) deficits in cerebellum and anterior cingulate of PTSD subjects [9] and the other using arterial spin labeling (ASL) sequence to find CBF changes in right parietal, frontal, and superior temporal cortices [10]. The DSC perfusion is minimally invasive and cannot be used for dynamic analysis. With the development of ASL technique, the dynamic ASL-fMRI demonstrates potential superiority in functional connectivity analysis compared to conventional blood oxygen level-dependent (BOLD) fMRI [11] and has been applied in clinical studies [12, 13]. Due to relatively low spatial resolution, CBF estimation from the ASL technique is usually affected by partial volume (PV) effects, especially for those image voxels near tissue interfaces. Thus, effective PV correction on ASL data shall improve the performance of static CBF and dynamic functional connectivity analyses.

The aim of this study was to investigate possible alterations in CBF and functional connectivity between survivors with recent onset PTSD and without PTSD, who experienced the same coal mining flood disaster, using the pulsed ASL (PASL) sequence. For better CBF estimation, the linear regression (LR) method was used to correct PV effects in PASL data [14]. Both uncorrected and PV-corrected CBF maps were used for CBF analysis to evaluate the gain of PV correction.

## Methods

### Subjects

All of the twenty subjects were right-hand males survived from a coal mining flood disaster occurred on July 29th, 2007, in Henan province of China. In the mining disaster, 69 miners were trapped for 72 hours, and all of them were rescued and survived. After that, 48 survivors were hospitalized and received a medical checkup. Six months later, 17 survivors of them met the diagnostic criteria for PTSD using DSM-IV [15] and the Structured Clinical Interview for DSM-IV (SCID) [16], and 10 agreed to participate in this MRI study as the PTSD group. The severity of their symptoms was assessed using the Chinese version of the Clinician-Administered PTSD Scale (CAPS) [17]. In addition, 10 out of 31 survivors without PTSD agreed to participate in the MRI study as the non-PTSD group. The elapsed time between the traumatic event and MRI scans ranged from 187 to 190 days.

With the recruited PTSD and non-PTSD groups in this study, each group had 10 male subjects. The age of PTSD group ranged from 31 to 50, while that of non-PTSD group ranged from 28 to 42, respectively. The identified PTSD subjects had never received any psychiatric treatment before. Moreover, none of the subjects had a history of treatment with psychotropic drugs or of substance (alcohol, smoking or drug) abuse. The study was conducted according to the principles in the Declaration of Helsinki and approved by the Institutional Board of the Fourth Military Medical University. All subjects received a comprehensive description of the MRI study and gave voluntarily written informed consent before entering the study.

### MRI Acquisition

All MRI scans were acquired on a 3.0T MR scanner (MAGNETOM Trio, Siemens AG, Erlangen, Germany). ASL perfusion images were acquired using a PASL sequence, with imaging parameters set as TR = 2700 ms, TE = 13 ms, slice thickness = 3.0 mm, acquisition matrix = 64×64, flip angle = 90°, field of view (FOV) = 224×224 mm, TI_2_ = 1800 ms, and slice = 25 with 1.5 mm gap. With the slice ascending, TI_2_ increases with a slice of 45 ms. Each ASL series contains 121 images, including M0 and 60 label/control pairs. Meanwhile, a 3D magnetization prepared rapid acquisition gradient echo (MPRAGE) T1-weighted sequence covering the whole-brain (176 sagittal slices) was acquired. Corresponding acquisition parameters were set as TR = 1900 ms, TE = 2.26 ms, TI = 900ms, flip angle = 9°, acquisition matrix = 256×256, FOV = 220 mm, and 1.00 mm slice thickness with no inter-slice gap.

### Preprocessing

The image analysis was performed on a computer workstation installed with MATLAB 7.11.0 under SPM8 (Wellcome Department of Imaging Neuroscience, London, UK; http://www.fil.ion.ucl.ac.uk/spm/software/spm8/).

For each subject, the PASL and structural images were preprocessed by SPM8 as follows: 1) Separate realignments of control and label image series were performed to correct head movement. Bad volumes were excluded with any of the following: (1) spike that was not in 2.5 standard deviations of the average variation; (2) abnormal head-motion with overall movement > 2mm or rotation > 2°; (3) abnormal head-motion with movement > 0.75 mm or rotation > 1.5° between TRs. 2) The corresponding structural image was segmented to generate posterior probability maps of gray matter (GM), white matter (WM) and cerebrospinal fluid (CSF), using the standard processing step of SPM. 3) The segmented structural image and the mean image generated from realigned PASL differences were co-registered to obtain the percentage of different-type tissues at each voxel of the perfusion map, based on transformation of structural and PASL coordinates with MNI coordinate. On this basis, the simple (pairwise) subtraction of label/control pairs was used to generate total sixty difference images.

### CBF Calculation

Since there is no specific tool in SPM8 for PV correction and CBF quantification, in this study, a custom-built Matlab package was developed, in which the well-established LR method was utilized to correct the PV effect [14] and the two compartment model was adopted to calculate the CBF maps [18]. The detailed information on PV correction and CBF quantification is shown in S1 Appendix.

For uncorrected CBF (No correction): The uncorrected CBF map was directly calculated with only GM or WM component, using the two-compartment model [18].

For PV-corrected CBF (PV correction): Considering relatively low spatial resolution of PASL data, a widely-used LR method was adopted to correct the PV effect in perfusion images [14]. The separate GM and WM contribution of each label/control difference image can be accurately obtained directly from the corrected data. Then, the PV-corrected CBF map can be calculated from the corrected contributions using the two-compartment model of ASL technique [14, 18].

In this study, a total of sixty label/control pairs of uncorrected or PV-corrected data were acquired for each participant and the resulting CBF maps constituted a CBF time series with all 3D spatial frames ordered along the time axis by the acquisition time. After each map of the CBF series was normalized to the MNI space and spatially smoothed with an 8-mm Gaussian kernel, all maps were averaged to generate the mean CBF map of the subject

### Statistical Analysis

The mean CBF map was used for CBF analysis between the PTSD and non-PTSD groups. Based on altered regions identified by CBF analysis, CBF time series were further used for functional connectivity analysis.

To demonstrate potential effects of PV correction on CBF analysis, both uncorrected and PV-corrected CBF maps from two groups were used in this study, respectively. For human beings, a remarkable CBF decrease could be observed during aging [19] and the two groups (PTSD and non-PTSD) differed significantly in age (Table 1). To account for the age effect, the group comparison of mean CBF maps was performed using two sample *t*-test with age as a covariate in SPM8. Two planned contrasts were examined as follows: PTSD>non-PTSD subjects and PTSD<non-PTSD subjects. The statistical significance level was set to an uncorrected *p* value of 0.001, with the cluster size larger than a minimum of 50 contiguous voxels. On this basis, all identified regions acquired in CBF analysis were selected as regions of interest (ROI), then ROI-based correlation analysis between the mean CBF of each identified ROI and the CAPS value was performed in patients with PTSD, by using Pearson's partial correlation analysis in SPSS 13.0. Here, age was also treated as a controlling covariate and the significance level was set as *p*<0.05.

**Table 1:** Subject physical and clinical characteristics.

	PTSD	non-PTSD	*t* tests	
			*t value*	*P*
Age (years)	40.80±6.83	34.30±5.37	2.318	0.032
Education level	6.67±2.06	7.25±2.38	1.72	0.14
CAPS^a^	78.72±17.2	31.40±18.57	6.306	<0.001

^a^ CAPS: Clinician-Administered PTSD Scale, with higher scores indicating greater PTSD severity.

Based on the results of PV-corrected CBF analysis, regions that exhibited significant CBF alterations in the PTSD group were selected as seed regions for functional connectivity analysis. The time series of all voxels within a seed region were then extracted from all CBF maps and averaged to obtain the reference time curve. Functional connectivity analysis was then performed by calculating the correlation between the reference curve and that of each voxel. After applying the Fisher’s *r*-to-*z* transform, group differences in functional connectivity were then investigated using a two-sample *t* test with age as the covariate. The statistical significance level was set to an uncorrected *p* value of 0.001, with the cluster size larger than a minimum of 50 contiguous voxels. ROI-based correlation analysis between the mean connectivity of identified ROI and the CAPS value was then performed in patients with PTSD using Pearson's partial correlation analysis in SPSS 13.0. Age was treated as a controlling covariate and the significance level was set as *p*<0.05.

**Fig 1:**
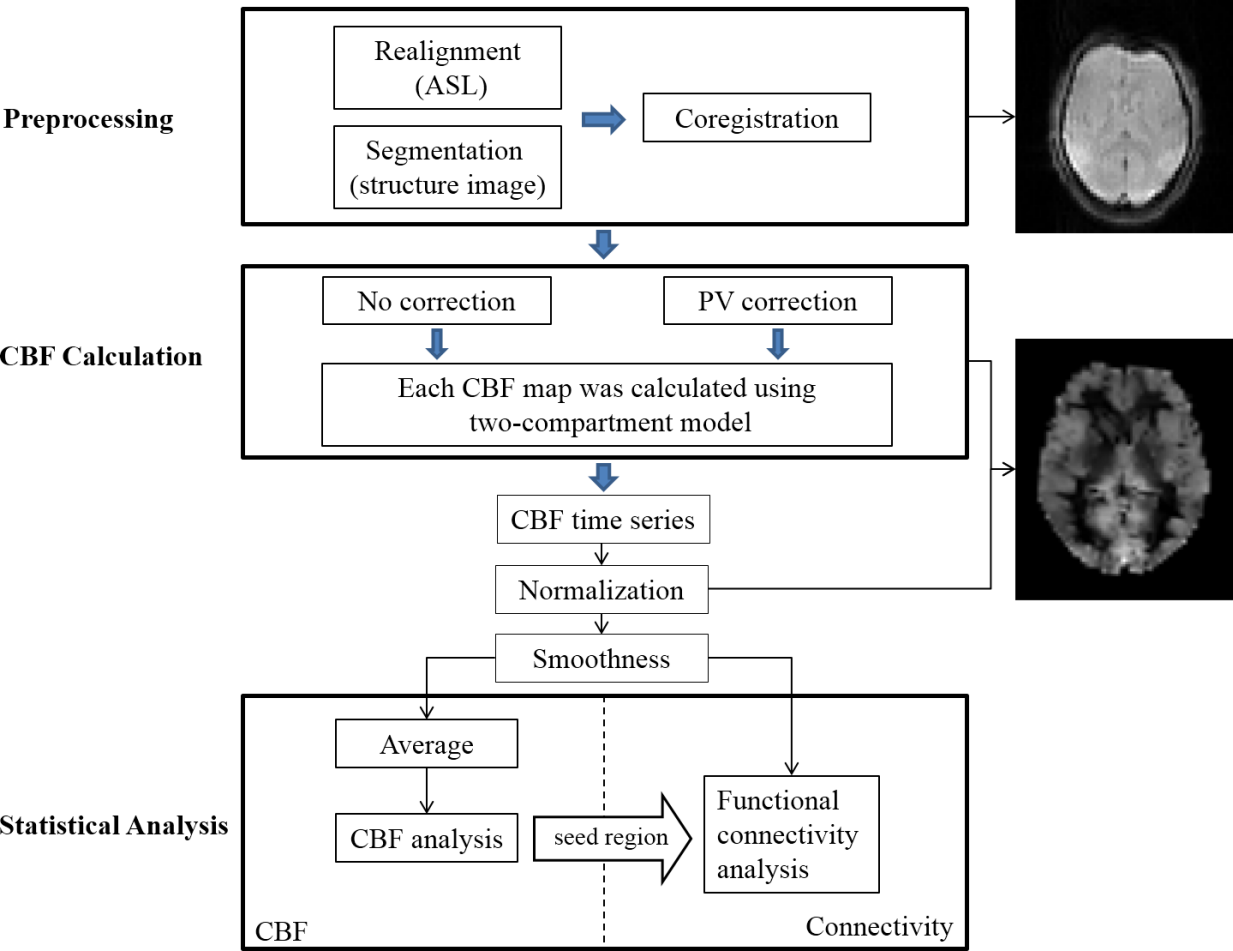
The processing and analysis procedure of the present study.

To better demonstrate the processing and analysis procedure of present study, a flowchart is depicted in Fig 1.

## Results

### Subject Demographics

Table 1 gives the mean and standard deviation of physical and clinical characteristics for the subjects. As expected, the PTSD group has higher CAPS scores than those of non-PTSD group. The two groups (PTSD and non-PTSD) significantly differed in age but no significant difference was found in the level of education.

### Group Difference with Uncorrected CBF

**Fig 2:**
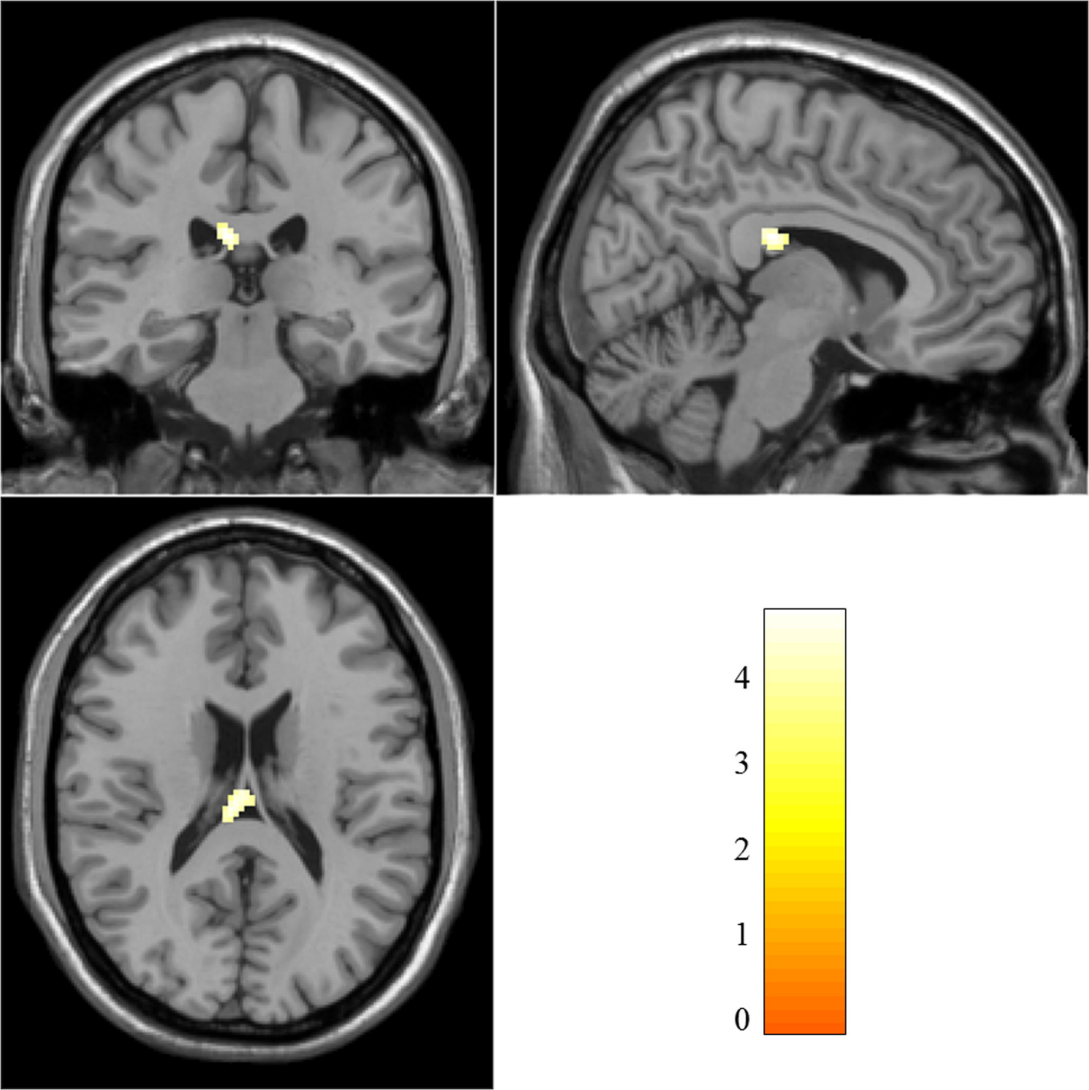
Significantly decreased CBF region of PTSD group compared with non-PTSD group. The two sample *t*-test was performed on uncorrected CBF maps of the PTSD and non-PTSD groups (*p*<0.001, uncorrected, with cluster size larger than a minimum of 50 contiguous voxels).

Based on CBF calculation, the uncorrected and PV-corrected CBF values of each subject were calculated, as shown in S2 Appendix. Comparisons of uncorrected CBF in survivors with and without PTSD demonstrate the CBF deficits in right corpus callosum of patients with PTSD (MNI: 10, –28, 24; cluster size: 59; *t* score: 4.7514), as shown in Fig 2.

### Group Difference with PV-corrected CBF

**Fig 3:**
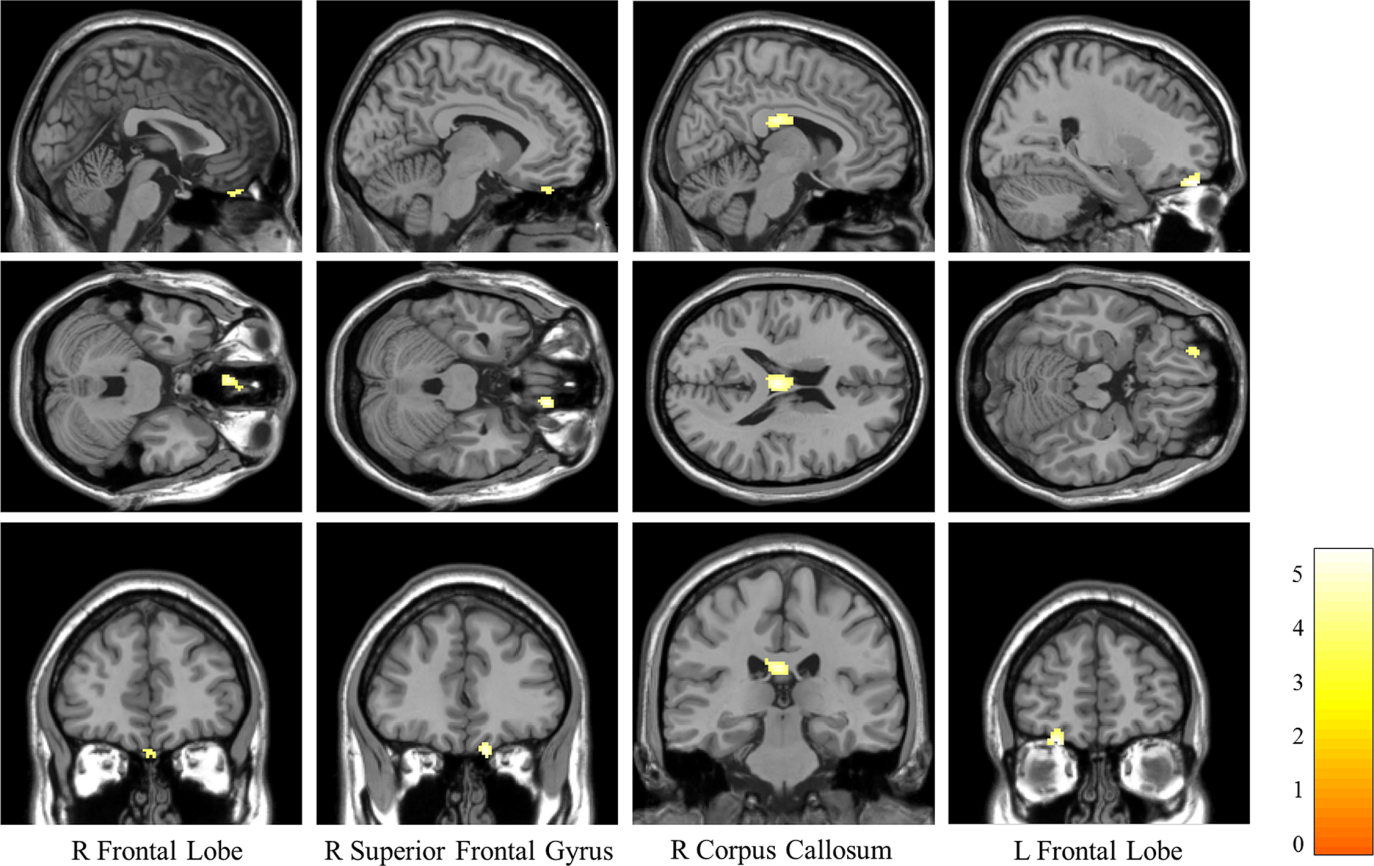
Significantly decreased CBF regions of PTSD group compared with non-PTSD group. The two sample *t*-test was performed on PV-corrected CBF maps of the PTSD and non-PTSD groups (*p*<0.001, uncorrected, with cluster size larger than a minimum of 50 contiguous voxels).

Comparisons of PV-corrected CBF in survivors with and without PTSD are shown in Table 2 and Fig 3. The results indicate that in the right frontal lobe, right superior frontal gyrus, right corpus callosum, and left frontal lobe, CBF values of subjects with PTSD were obviously lower than those without PTSD.

**Table 2:** Significantly decreased CBF regions of PTSD group compared with non-PTSD group.

Brain regions	MNI (x,y,z)	Cluster size	*t* score
R frontal lobe	6, 38, –30	115	5.4019
R superior frontal gyrus	26, 52, –22	94	5.2943
R corpus callosum	26, –22, 22	189	5.3209
L frontal lobe	–12, 40, 28	70	5.18

### Correlation with Symptom Severity

**Fig 4:**
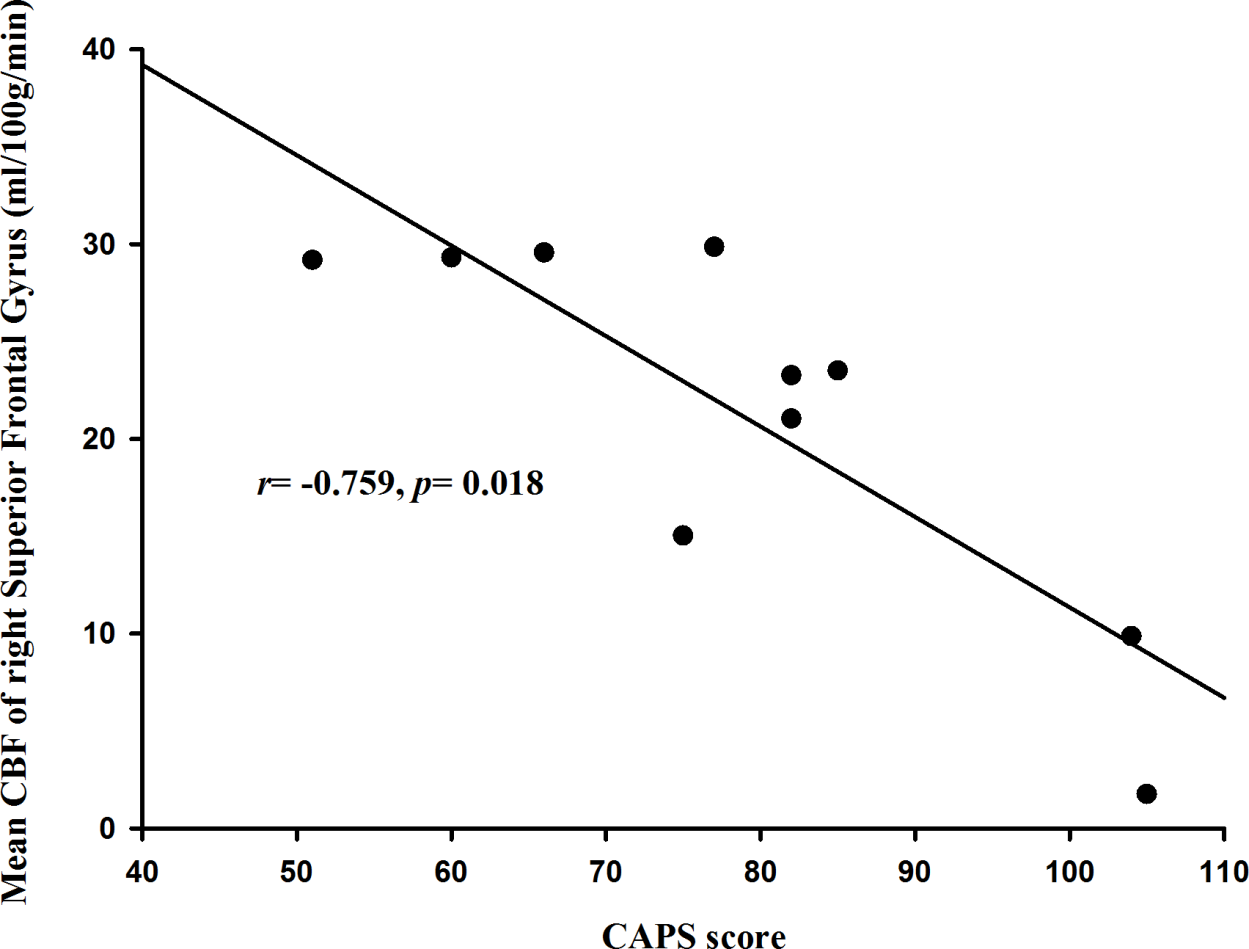
Mean CBF of the right superior frontal gyrus correlated negatively with the CAPS scores of PTSD patients when using Pearson's partial correlation analysis with age as a controlling covariate, *p*<0.05.

Among four regions identified by PV-corrected CBF analysis, only the mean CBF of the right superior frontal gyrus correlated negatively with the CAPS score (*r* = −0.759, *p* = 0.018), as shown in Fig 4. This result indicates that the decrease of CBF in the right superior frontal gyrus was associated with the symptom severity of the disorder (CAPS score) in subjects with PTSD.

### Functional Connectivity Analysis

**Fig 5:**
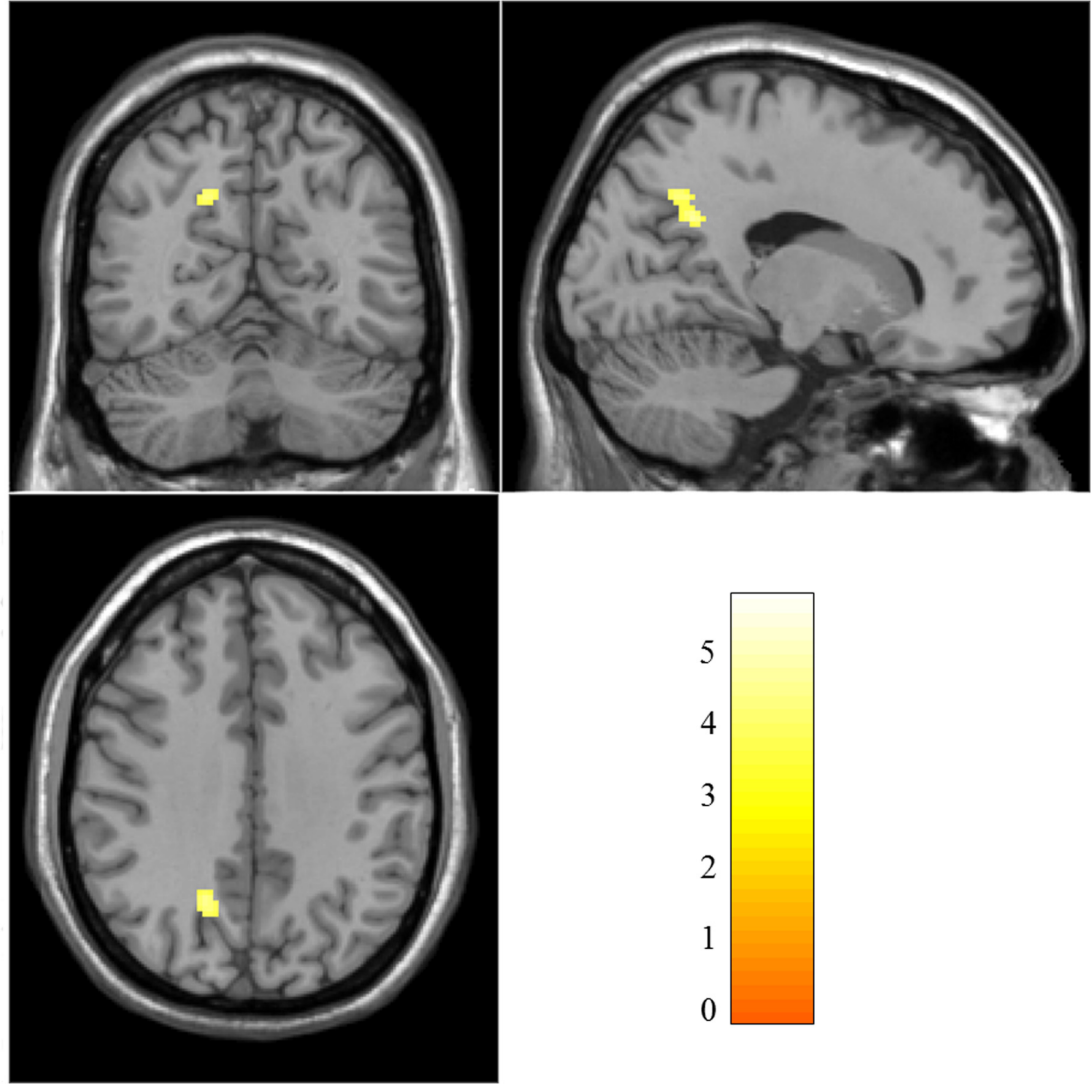
Increased functional connectivity between the left frontal lobe and the right parietal lobe in the PTSD group. The two sample *t*-test was performed on connectivity strengths of the PTSD and non-PTSD groups (*p*<0.001, uncorrected, with cluster size larger than a minimum of 50 contiguous voxels).

Based on the results of CBF analysis with PV-corrected maps, each cluster that exhibited significant CBF alteration in the PTSD group was selected as the seed region for the functional connectivity analysis. However, only the left frontal lobe showed alteration of functional connectivity with other brain region using resting-state functional connectivity analysis. Increased functional connectivity between the left frontal lobe and the right parietal lobe (MNI: 18, –54, 28; cluster size: 88; *t* score: 5.8119) was observed in the PTSD group (Fig 5). In this cluster, 78 out of 88 voxels are in precuneus. With the transformation provided by Talairach client, the peak MNI is also located in the precuneus. Thus, precuneus is also identified as a significantly increased functional connectivity region with left frontal lobe. On this basis, the correlation between mean connectivity of the voxels in the right parietal lobe (precuneus) and the CAPS score was also calculated, but no significant correlation was found.

## Discussion

The present study investigated differences in whole-brain CBF maps and functional connectivity of survivors with and without recent onset PTSD from a coal mining flood disaster, using the PASL sequence. The major contributions of this study are in two aspects: First, we found some symptom-specific CBF and functional connectivity alterations in PTSD patients. Second, the introduction of PV correction into the processing pipeline of the ASL technique could benefit further CBF and connectivity analysis.

In this study, we found CBF deficits in the bilateral frontal lobe, right superior frontal gyrus, and right corpus callosum. The frontal lobe is thought to play an important role in human memory formation [20] and longer term memory maintaining [21], while the superior frontal gyrus, which is located in the frontal lobe, contributes to higher cognitive functions and particularly to working memory [22]. A perfusion study using SPECT showed decreased perfusion in the superior frontal gyrus of PTSD patients [23]. Meanwhile, fMRI studies have demonstrated that PTSD patients presented increased functional connectivity between the posterior cingulate cortex and the right superior frontal gyrus [24] as well as enhanced regional homogeneity in the left inferior parietal lobule and right superior frontal gyrus [25]. In our previous volumetric analysis, GM deficit in right frontal lobe was identified to associate with the symptom severity of these trauma survivors [26]. Further analysis on cortical thickness found cortical thinning in the right inferior frontal gyrus [1], which is also located in the frontal lobe. With the new findings of CBF deficit in this study, our series analyses have suggested the importance of memory-related frontal lobe to the recent onset PTSD after a single prolonged trauma exposure. Though the trend of alterations may be different due to sample size, PTSD subtype, and analysis method, these aberrant regions were all located in the frontal lobe, supporting the importance of memory-related frontal lobe to PTSD.

The corpus callosum lies immediately below the cingulate gyrus, connecting the two hemispheres of the brain. An atrophy of corpus callosum was identified in veterans [27] and children [19] with PTSD. Moreover, several DTI studies found reduced fractional anisotropy in corpus callosum, indicating less connectivity [20, 28]. In addition, it is reported that the volume of corpus callosum is related to the improvement of working memory ability and transcallosal pathways may be important in the recovery of cognitive functions [29]. It should be mentioned that as an inherently low resolution and low signal to noise ratio (SNR) technique, ASL data faces some challenges to perform group-wise comparisons in deep WM ASL measurements. In this study, most WM CBF values including that of the identified region in the corpus callosum were quite low, close to that of the ventricles. We will further discuss the problem in the limitation of this study.

For better CBF estimation from low resolution perfusion data, the LR method was used to correct the PV effect in this study. CBF analysis results shown in Fig 2 and Fig 3 demonstrate that using uncorrected CBF maps, only the perfusion deficit in right corpus callosum was identified. With the use of PV-corrected CBF maps, three more regions (bilateral frontal lobes and right superior frontal gyrus) were further identified, where most of them located at boundary regions between GM and other tissues. Based on the report from xjview (http://www.alivelearn.net/xjview8/), the clusters shown in Fig 3 were all mixed regions of GM and WM. These findings indicate that the PV-corrected CBF map can reflect more subtle perfusion changes, especially at tissue interfaces, which may be beneficial to further perfusion and connectivity analysis. It’s an attempt to address the challenges that ASL faces for static and dynamic analyses and also emphasizes the need for the inclusion of PV correction in the ASL processing pipeline.

It is worth noting that some of identified regions from CBF analysis using PV-corrected CBF are at the border of brain (Fig 2) and some PTSD patients have relatively low regional CBF values (Fig 4). Since the identified region is at the border of brain, the low regional CBF may reflect artifacts. Considering that the subjects with low regional CBF have relatively high CAPS scores (Fig 4), low regional CBF may be also caused by severe degree of PTSD. Due to small sample size of this study, this assumption is difficult to be effectively validated. Meanwhile, the small sample size may also result in the weak statistics, thus we added it in the limitation.

Functional connectivity analysis has been demonstrated to be a powerful approach to identify biomarkers for different brain diseases [30]. While most studies used BOLD-fMRI data, functional connectivity analysis using ASL-fMRI is just starting out. In this study, based on identified regions of CBF analysis, we used PASL data and found increased resting-state functional connectivity of left frontal lobe with right parietal lobe (precuneus). The parietal lobe is thought to be involved in temporal and spatial orientation function [31], which may participate in processing of spatial and temporal information related to a traumatic event [32]. The observation of diminished activation in the parietal lobes during traumatic memory retrieval may provide an explanation for why traumatic memories are experienced as being ‘present tense’ [32]. Currently, no study has reported the alteration of functional connectivity between the frontal lobe and parietal lobe (precuneus) in PTSD patients, however, decreased connectivity from the frontal lobe to the parietal lobe was identified in patients with cognitive impairment [33]. Increased connectivity between left frontal lobe and precuneus might be related to processing internal thoughts and self-referent, which was recently identified in borderline personality disorder (BPD) [34]. PTSD is an anxiety disorder, which is sometimes accompanied with mild cognitive impairment and/or BPD. Thus, the reports about cognitive impairment and BPD may indicate the importance of this connectivity alteration for PTSD. Moreover, it's worth noting that the precuneus is a key region of the default mode network (DMN), which is reported to integrate information from recent experience with past memories [35]. In general, DMN is believed to “reflect neural functions that consolidate the past, stabilize brain ensembles, and prepare us for the future” [36], thus the PTSD-specific alteration in DMN may be benefit to processing the traumatic memories [37]. Increased functional connectivity between the left frontal lobe and precuneus observed in this study and cortical thinning in precuneus found in our previous study [1] may indicate connectivity alteration within DMN for patients with PTSD.

Several limitations of this study should be addressed. Firstly, the sample size is small. The results of CBF and functional connectivity analyses could not survive after multiple comparison correction, which may be due to the small sample size used in this study. For subjects surviving from a coal mining flood disaster, we could not control the sample size, just like some studies on PTSD induced by other sudden disasters, such as a fire disaster [38] or sarin attacks [4]. However, we’ve tried our best to make all data consistent to alleviate possible sampling bias. Secondly, as indicated above, CBF-based analysis faces challenges in performing group-wise comparisons in deep WM ASL measurements due to relatively low resolution and low SNR of ASL data. In this study, PV correction was included as an attempt to improve the resolution, which can also suppress noise inherently [14] and have demonstrated promising results. Further studies on the improvement of CBF measurements, such as the improvement of SNR, shall be conducted for better CBF calculation and group-wise analysis.

## Supporting Information

S1 Appendix Processing steps for PV correction and CBF quantification. (DOC)

S2 Appendix PV-corrected and uncorrected CBF values. (DOC)
