# Supplementary material for: Perfusion Deficits and Functional Connectivity Alterations in Memory-Related Regions of Patients with Post-Traumatic Stress Disorder
Source: PLoS One. 2016 May 23;11(5):e0156016. doi: 10.1371/journal.pone.0156016 (PMC4877105; doi:10.1371/journal.pone.0156016)
Supplement: S1 Appendix — (DOC) [file pone.0156016.s001.doc]

**Supporting Information**

**S1 Appendix. Processing steps for PV correction and CBF quantification**

In this study, we developed a custom-built Matlab package in which the well-established LR method was utilized to correct the PV effect [1] and the two-compartment model was adopted to calculate the CBF maps [2]. The perfusion model and processing steps are listed below.

**1. Voxel-wise perfusion model**

In the current CBF quantitative method, the CBF *f* can be obtained as:

$f_{tissue}=(\frac{\Delta M}{M_{0}})F_{tissue}$ (1)

where *F_tissue_* is a tissue-specific parameter, and *M*_0_ represents the equilibrium brain tissue magnetization obtained from M0 image.

Due to low spatial resolution in PASL images, the label-control difference signal (∆*M*) and M0 image were usually assumed to include mixed contributions of gray matter (GM), white matter (WM), and cerebrospinal fluid (CSF). To differentiate the contributions of GM, WM and CSF to blood flow, the CBF can be described as:

$\text{CBF}=P_{GM}(\frac{\Delta M_{GM}}{M_{0GM}})F_{GM}+P_{WM}(\frac{\Delta M_{WM}}{M_{0WM}})F_{WM}+P_{CSF}(\frac{\Delta M_{CSF}}{M_{0CSF}})F_{CSF}$ (2)

In Eq (2), *P_GM_*, *P_WM_*, and *P_CSF_* represent the probabilities of GM, WM and CSF tissues, which can be obtained from T1 weighted images of the same subject. Typically, no perfusion signal should arise from CSF [3], thus, ∆*M_CSF_* = 0. The five parameters, i.e., *M_0GM_*, *M_0WM_*, *M_0CSF_*, ∆*M_GM_*, and ∆*M_WM_*, can be estimated using the LR method [1], as described in section 2. The *F_GM_* and *F_WM_* can be calculated using two-compartment model [2], as described in section 3.

**2. PV correction using the LR method**

The PV correction involves two steps. The first step estimates *M_0GM_*, *M_0WM_*, and *M_0CSF_* from the M0 image. The second step estimates ∆*M_GM_* and ∆*M_WM_* from the label-control difference image.

Step 1: Assuming that *M_0GM_*, *M_0WM_*, and *M_0CSF_* are approximately constant in a local *n*×*n*×1 region, *M_0GM_*, *M_0WM_*, and *M_0CSF_* of the region can be estimated using the LR method:

$\overline{M_{0j}}=(P^{T}P)^{-1}P^{T}\overline{M_{0}}$ (3)

where *j* corresponds to GM, WM or CSF, *P* is a *n*^2^×3 matrix formed by stacking the individual *P_GM_*, *P_WM_*, and *P_CSF_* of each voxel in the regression region, and $\overline{M_{0}}$ is a column vector with *n*^2^ M0 signals in the regression region.

Step 2: Assuming that ∆*M_GM_* and ∆*M_WM_* are approximately constant in a local *n*×*n*×1 region, ∆*M_GM_* and ∆*M_WM_* of the region can be estimated using the LR method:

$\overline{\Delta M_{j}}=(P^{T}P)^{-1}P^{T}\overline{\Delta M}$ (4)

where *j* corresponds to GM or WM, *P* is a *n*^2^×2 matrix formed by stacking the individual *P_GM_* and *P_WM_* of each voxel in the regression region, and $\overline{\Delta M}$ is a column vector with *n*^2^ difference signals in the regression region.

In this study, we used 5×5×1 regression kernel, which was suggested to provide the best compromise between smoothing and PV correction [1].

**3. CBF quantification**

For PASL, *F_tissue_* can be calculated by:

$\begin{matrix} F_{tissue}=\frac{\lambda}{2\alpha}\left\{\begin{matrix} e^{-TI_{2}/T_{1app}}[e^{\min[TI_{2},\delta+TI_{1}](T_{1a}-T_{1app})/(T_{1a}T_{1app})}-e^{\delta(T_{1a}-T_{1app})/(T_{1a}T_{1app})}] \\ (T_{1a}T_{1app})/(T_{1a}-T_{1app})+e^{-TI_{2}/T_{1a}}[(\min[\delta a-\Delta TI,0]-\delta a) \\ -(\min[\delta-\Delta TI,0]-\delta)] \end{matrix}\right\}^{-1} TI_{2}>\delta\\ F_{tissue}=-\frac{\lambda}{2\alpha}e^{TI_{2}/T_{1a}}(\min[\delta a-\Delta TI,0]-(\delta a-TI_{2}))^{-1} TI_{2}<\delta\end{matrix}$ (5)

Parameter notations and corresponding values were given in the following table.

| Parameter | Description | Values | References |
| --- | --- | --- | --- |
| *T*_1app_ | *T*_1_ of brain tissue | 1240 ms | [2] |
| *T*_1a_ | *T*_1_ of blood | 1490 ms | [2] |
| *λ* | blood/tissue water partition coefficient | 0.95ml/g (GM)  0.82ml/g (WM) | [4] |
| *α* | inversion efficiency | 0.95 | [5] |
| *δα* | 112.5 ms/slice with a starting point for the first slice of | 200 ms (GM)  420 ms (WM) | [6] |
| *δ* | transit time from the labeling region to the tissue compartment | 500 ms (GM)  700 ms (WM) | [1] |

**References**

1. Asllani I, Borogovac A, Brown TR. Regression algorithm correcting for partial volume effects in arterial spin labeling MRI. Magn Reson Med. 2008;60(6):1362-71. Epub 2008/10/02. doi: 10.1002/mrm.21670. PubMed PMID: 18828149.

2. Wang J, Alsop DC, Li L, Listerud J, Gonzalez-At JB, Schnall MD, et al. Comparison of quantitative perfusion imaging using arterial spin labeling at 1.5 and 4.0 Tesla. Magn Reson Med. 2002;48(2):242-54. Epub 2002/09/05. doi: 10.1002/mrm.10211. PubMed PMID: 12210932.

3. Golay X, Hendrikse J, Lim TC. Perfusion imaging using arterial spin labeling. Top Magn Reson Imaging. 2004;15(1):10-27. Epub 2004/04/02. doi: 00002142-200402000-00003 [pii]. PubMed PMID: 15057170.

4. Herscovitch P, Raichle ME. What is the correct value for the brain--blood partition coefficient for water? Journal of cerebral blood flow and metabolism : official journal of the International Society of Cerebral Blood Flow and Metabolism. 1985;5(1):65-9. doi: 10.1038/jcbfm.1985.9. PubMed PMID: 3871783.

5. Wang Y, Saykin AJ, Pfeuffer J, Lin C, Mosier KM, Shen L, et al. Regional reproducibility of pulsed arterial spin labeling perfusion imaging at 3T. Neuroimage. 2011;54(2):1188-95. doi: 10.1016/j.neuroimage.2010.08.043. PubMed PMID: 20800097; PubMed Central PMCID: PMC2997151.

6. Asllani I, Borogovac A, Wright C, Sacco R, Brown TR, Zarahn E. An investigation of statistical power for continuous arterial spin labeling imaging at 1.5 T. Neuroimage. 2008;39(3):1246-56. doi: 10.1016/j.neuroimage.2007.10.015. PubMed PMID: 18036834; PubMed Central PMCID: PMC2665307.
