# Supplementary material for: Perfusion Deficits and Functional Connectivity Alterations in Memory-Related Regions of Patients with Post-Traumatic Stress Disorder
Source: PLoS One. 2016 May 23;11(5):e0156016. doi: 10.1371/journal.pone.0156016 (PMC4877105; doi:10.1371/journal.pone.0156016)
Supplement: S2 Appendix — (DOC) [file pone.0156016.s002.doc]

**Supporting Information**

**S2 Appendix. PV-corrected and uncorrected CBF values**

Table 1. PV-corrected GM CBF, WM CBF, and the uncorrected CBF values (mean±std).

|  | PV-corrected GM CBF | PV-corrected WM CBF | uncorrected CBF |
| --- | --- | --- | --- |
| PTSD | 50.76±19.47 | 18.61±13.05 | 41.95±26.76 |
|  | 54.50±22.95 | 21.37±16.01 | 45.63±37.58 |
|  | 49.40±21.93 | 19.12±16.39 | 41.67±26.49 |
|  | 49.93±19.57 | 16.47±13.08 | 41.27±27.77 |
|  | 52.47±25.41 | 17.51±13.62 | 42.35±32.28 |
|  | 66.28±28.73 | 24.27±17.41 | 48.75±33.32 |
|  | 52.74±19.43 | 17.51±14.12 | 40.33±26.16 |
|  | 63.22±24.83 | 17.39±15.86 | 50.33±34.32 |
|  | 48.61±13.48 | 16.66±11.01 | 40.75±31.91 |
|  | 47.87±15.00 | 16.64±10.59 | 39.39±34.43 |
| control | 63.97±25.65 | 20.98±18.66 | 52.92±30.28 |
|  | 53.11±15.16 | 18.84±13.45 | 41.26±29.07 |
|  | 56.18±19.86 | 22.61±15.21 | 47.28±26.78 |
|  | 57.75±26.03 | 19.51±13.26 | 45.91±39.47 |
|  | 52.72±19.42 | 18.81±14.18 | 42.46±28.17 |
|  | 57.89±19.95 | 18.76±10.06 | 50.70±38.39 |
|  | 53.24±19.01 | 15.13±12.27 | 49.81±34.51 |
|  | 50.99±16.57 | 17.79±6.52 | 41.20±19.98 |
|  | 62.15±22.85 | 27.03±21.89 | 54.63±26.51 |
|  | 56.31±19.28 | 18.89±10.07 | 47.68±30.95 |
